# Predicting severe intraventricular hemorrhage in very preterm and/or very low birth weight infants: a nomogram approach

**DOI:** 10.3389/fped.2026.1838932

**Published:** 2026-06-03

**Authors:** Wenying Meng, Yang Li, Xiaowei Sun, Jialin Wen, Haiping Yang

**Affiliations:** 1 Department of Pediatrics, Cangzhou Central Hospital, Cangzhou, Hebei, China; 2 Department of Pediatrics, Qilu Hospital, Shandong University, Jinan, Shandong, China; 3 Department of Pediatrics, Beijing Friendship Hospital, Capital Medical University, Beijing, China

**Keywords:** intraventricular hemorrhage, predictive model, preterm birth, risk factors, very low birth weight (VLBW) infants

## Abstract

**Background:**

Severe intraventricular hemorrhage (IVH) is a major brain injury in very preterm and/or very low birth weight infants. We developed a simple model to distinguish severe IVH from mild IVH among very preterm and/or very low birth weight infants with IVH using routinely available perinatal factors.

**Methods:**

This retrospective multicenter study included infants born at <32 weeks’ gestation and/or with birth weight < 1,500 g admitted to three tertiary neonatal intensive care units (NICUs) in northern China (January 2023–December 2024). IVH was assessed by cranial ultrasonography and graded by the modified Papile system; grades III–IV were defined as severe. Least absolute shrinkage and selection operator (LASSO) and multivariable logistic regression were used to build a nomogram. Discrimination and calibration were evaluated, internal validation was performed using bootstrap resampling, and decision curve analysis was used as an exploratory assessment of clinical utility.

**Results:**

Among 161 infants with IVH, gestational age, base excess (BE), and meningitis-consistent cerebrospinal fluid (CSF) abnormalities were retained as predictors. The apparent C-index/AUC was 0.744 (95% CI: 0.653–0.834), and the optimism-corrected C-index/AUC after bootstrap internal validation was 0.727 (95% CI: 0.646–0.826). The Brier score was 0.171, and the optimism-corrected calibration slope was 0.934.

**Conclusions:**

Among very preterm and/or very low birth weight infants with IVH, a nomogram based on gestational age, BE, and meningitis-consistent CSF abnormalities may help preliminary risk stratification and identify infants who may warrant closer cranial ultrasound surveillance and monitoring. External validation is required before broader clinical implementation.

## Introduction

Severe neonatal intraventricular hemorrhage (IVH) is one of the most common forms of brain injury in the early neonatal period (1). Reported mortality for severe IVH varies widely (approximately 55.2%) (2), and survivors frequently experience lasting neurological sequelae, such as cerebral palsy, epilepsy, motor disability, and cognitive impairment (3–5). With ongoing improvements in neonatal intensive care, increasing numbers of infants born at very low gestational ages now survive, expanding the population vulnerable to IVH and its long-term consequences (6). In this study, we analyzed very preterm and/or very low birth weight infants admitted to the neonatal intensive care units (NICUs) of three tertiary hospitals in northern China. Our objective was to develop a predictive model to distinguish severe IVH from mild IVH among very preterm and/or very low birth weight infants with IVH, with the aim of facilitating earlier risk stratification and closer monitoring.

## Methods

### Data collection and diagnosis

Between January 2023 and December 2024, we enrolled infants born at <32 weeks' gestation and/or with a birth weight < 1,500 g who were admitted to the neonatal intensive care units of Qilu Hospital of Shandong University, Beijing Friendship Hospital (Capital Medical University), and Cangzhou Central Hospital. All participants were delivered in the obstetric departments of the three centers and were transferred directly to the neonatal unit immediately after birth.

Participants were excluded if they were outborn and referred from another institution, had missing or insufficient records for key predictors or outcome assessment, or had active treatment withheld or withdrawn before completion of the early outcome surveillance.

This study was conducted in accordance with the Declaration of Helsinki and was approved by the Ethics Committee of Cangzhou Central Hospital (Approval No. 2026–026-01). Owing to the retrospective nature of the study, the requirement for informed consent was waived.

### Risk factors

We obtained detailed perinatal information, covering both maternal health status and neonatal clinical characteristics. Maternal and antenatal variables included maternal age, mode of delivery, exposure to antenatal corticosteroids, hypertensive disorders of pregnancy, gestational diabetes, chorioamnionitis, premature rupture of membranes (PROM), use of assisted reproductive technology (ART), meconium-stained amniotic fluid (MSAF), and multiple gestation. These variables were selected to capture major obstetric and antenatal inflammatory conditions potentially associated with early-onset infection and IVH. However, more detailed inflammatory indicators, including maternal intrapartum fever, histological chorioamnionitis, funisitis, and placental pathological findings, were not consistently available across all three centers and therefore were not included as candidate predictors.

Neonatal characteristics included gestational age at birth, birth weight, sex, and 1-, 5-, and 10 min Apgar scores. Early respiratory support variables included postnatal pulmonary surfactant use and endotracheal intubation, recorded only if performed before or no later than the first cranial ultrasound diagnosis of severe IVH. Early laboratory indicators obtained within the first 24 h after birth included procalcitonin (PCT), white blood cell (WBC) count, neutrophil (NEU) count, lymphocyte (LYM) count, red blood cell (RBC) count, hemoglobin (Hb), platelet (PLT) count, and C-reactive protein (CRP). Arterial blood gas variables included pH, partial pressure of oxygen (PaO₂), partial pressure of carbon dioxide (PaCO₂), and base excess (BE). For these variables, the first arterial blood gas result obtained after NICU admission and within 24 h after birth was used in the analysis. Biochemical measurements included alanine aminotransferase (ALT), aspartate aminotransferase (AST), albumin (ALB), blood urea nitrogen (BUN), creatinine (CRE), creatine kinase (CK), and creatine kinase-MB (CKMB), using the earliest available results after NICU admission.

We also recorded major neonatal morbidities, including meningitis-consistent cerebrospinal fluid (CSF) abnormalities, early-onset sepsis (EOS), neonatal respiratory distress syndrome (NRDS), and patent ductus arteriosus (PDA). Because blood culture results are not available in real time, eligibility and the initial diagnosis of EOS in this study were based on a clinical diagnosis framework. Specifically, EOS was operationally defined as compatible clinical manifestations within 72 h of life plus ≥2 abnormal findings on nonspecific blood tests, including WBC count, absolute neutrophil count, immature-to-total neutrophil ratio, CRP, and PCT interpreted using postnatal-age–specific thresholds (Supplementary Table S1). This operational definition was used to guide further evaluation, including lumbar puncture when clinically feasible. Lumbar puncture was not performed as a routine screening procedure in all infants. It was considered only in infants with clinically suspected or clinical EOS and was performed when the infant was clinically stable enough to tolerate the procedure. If an infant was initially unstable, lumbar puncture was deferred until cardiorespiratory status had stabilized. Infants without clinical suspicion of EOS were not considered to have an indication for CSF analysis. Blood culture results were used retrospectively to classify confirmed EOS, whereas CSF findings were not used to determine EOS eligibility, thereby reducing incorporation bias.

Meningitis-consistent CSF abnormalities were defined as either a positive CSF culture or CSF findings suggestive of bacterial meningitis, including CSF white blood cell count ≥ 20/mm^3^ with >50% polymorphonuclear leukocytes, together with hypoglycorrhachia defined as CSF glucose < 2.2 mmol/L or CSF-to-serum glucose ratio < 0.40, with or without markedly elevated CSF protein (>1,880 mg/L). This variable was defined only on the basis of documented CSF findings; infants without CSF analysis were not considered to meet this definition unless predefined CSF biochemical/cytological or culture criteria were documented.

The primary outcome was severe IVH, defined as modified Papile grade III–IV IVH detected during the early postnatal cranial ultrasound surveillance window. Cranial ultrasound examinations were performed with a color Doppler system on postnatal days 1–3 and 5–7 to determine whether IVH had occurred and to assess its extent. IVH was graded using the modified Papile criteria: grade I, hemorrhage confined to the subependymal region; grade II, intraventricular bleeding occupying <50% of the ventricular volume; grade III, intraventricular bleeding accompanied by ventricular dilatation; and grade IV, hemorrhage with parenchymal involvement. For analysis, grades I–II were grouped as mild IVH, whereas grades III–IV were considered severe IVH (7). The diagnostic criteria for EOS are provided in Supplementary Table S1, and the definitions and timing of key variables are summarized in Supplementary Table S2. To preserve temporal ordering, only predictors measured before or no later than the first cranial ultrasound diagnosis of severe IVH were considered eligible for model development. For meningitis-consistent CSF abnormalities, the timing of lumbar puncture and cranial ultrasound was reviewed, and CSF abnormalities documented after the diagnosis of severe IVH were not used to define this predictor.

### Statistical analysis and model development

All analyses were performed in R (version 4.1.2). Missing data were handled using complete-case analysis. Infants with missing or insufficient records for candidate predictors or outcome assessment were excluded before model development, and no imputation was performed. All eligible variables listed in Table 1 that satisfied the temporal-ordering criterion were considered candidate predictors and were initially entered into the least absolute shrinkage and selection operator (LASSO) regression model. A total of 41 candidate predictors, including maternal and antenatal variables, neonatal clinical characteristics, early laboratory indicators, blood gas parameters, biochemical measurements, and major neonatal morbidities, were evaluated. Continuous variables were entered as continuous predictors, and categorical variables were coded as binary indicators where appropriate. The penalty parameter (*λ*) was selected by cross-validation. Variables with nonzero coefficients in the LASSO model were subsequently entered into a multivariable logistic regression model using the rms package to construct the final prediction model, which was visualized as a nomogram. Center was not included as an additional predictor or random effect in the final model because of the limited sample size and number of severe IVH events, which could further destabilize the model. This variable-selection strategy was used to reduce the risk of overfitting before multivariable model construction. Internal validation was performed using bootstrap resampling with 500 repetitions to estimate model optimism and obtain bias-corrected calibration. Apparent discrimination was reported using the C-index/AUC, and calibration was presented using both apparent and bias-corrected calibration curves. Because no independent external validation cohort was available, all performance estimates should be interpreted as internally validated exploratory estimates rather than evidence of external generalizability. Model performance was evaluated using discrimination, calibration, and overall accuracy. Discrimination was assessed using the apparent and optimism-corrected C-index/AUC. The 95% confidence interval for the optimism-corrected C-index/AUC was estimated using bootstrap resampling. Calibration was assessed using calibration intercept, calibration slope, and apparent and bias-corrected calibration curves. Overall accuracy was assessed using the Brier score. Decision curve analysis and the clinical impact curve were performed as exploratory analyses in the development cohort to describe potential clinical utility across threshold probabilities; these analyses were not considered evidence of clinical implementation readiness. A two-tailed *P* value < 0.05 was considered statistically significant.

**Table 1:** General characteristics of included preterm infants with IVH (*N* = 161).

Variable	Severe IVH group (*n* = 49)	Mild IVH group (*n* = 112)	χ^2^/Z value	*P* value
Sex (male), *n* (%)	28 (57.14)	63 (56.25)	0.011	0.916
Gestational age (Weeks)	28.00 (26.00, 29.00)	29.00 (28.00, 30.00)	−3.349	0.001
Birth weight (g)	1,100.00 (785.00, 1,225.00)	1,185.00 (1,000.00, 1,340.00)	−2.426	0.015
Apgar 1 min	6.00 (5.00, 9.00)	7.00 (6.00, 9.00)	−1.652	0.098
Apgar 5 min	9.00 (8.00, 10.00)	9.00 (8.00, 10.00)	−1.431	0.152
Apgar 10 min	9.00 (8.00, 10.00)	9.00 (8.00, 10.00)	−0.737	0.461
Postnatal administration of pulmonary surfactant, *n* (%)	48 (97.96)	98 (87.50)	4.414	0.036
Implementation of endotracheal intubation, *n* (%)	47 (95.92)	98 (87.50)	2.699	0.100
pH	7.24 (7.16, 7.34)	7.28 (7.22, 7.34)	2.163	0.032
Partial pressure of carbon dioxide (mmHg)	48.00 (37.50, 59.50)	49.00 (38.10, 57.00)	0.274	0.784
Partial pressure of oxygen (mmHg)	75.00 (56.40, 89.00)	80.50 (61.18, 106.00)	−1.631	0.103
Base excess (mmol/L)	−5.90 (−9.40, −4.30)	−4.25 (−6.95, −2.63)	−3.434	0.001
White blood cells (×10^9^/L)	6.65 (4.44, 12.41)	6.76 (4.06, 11.90)	0.312	0.755
Neutrophil (×10^9^/L)	4.28 (2.45, 8.33)	4.04 (1.89, 7.41)	0.749	0.454
Lymphocyte (×10^9^/L)	1.57 (0.85, 3.09)	1.94 (1.13, 3.01)	−0.975	0.329
Red blood cells (×10^12^/L)	3.95 (3.46, 4.36)	4.20 (3.72, 4.65)	−2.114	0.034
Hemoglobin (g/L)	154.00 (126.00, 172.50)	160.00 (144.00, 179.00)	2.339	0.021
Blood platelet count (×10^9^/L)	200.00 (138.50, 239.00)	177.50 (112.75, 237.75)	0.364	0.716
C-reactive protein (mg/L)	5.71 (0.75, 23.43)	7.47 (0.34, 17.28)	0.760	0.448
Procalcitonin (ng/mL)	9.29 (1.25, 32.05)	3.21 (0.43, 16.05)	2.201	0.028
Alanine aminotransferase (U/L)	5.00 (3.00, 6.00)	4.00 (3.00, 6.00)	1.276	0.202
Aspartate aminotransferase (U/L)	60.00 (38.10, 85.50)	48.95 (37.00, 64.95)	1.924	0.054
Serum albumin (g/L)	26.90 (25.45, 29.70)	28.25 (25.83, 31.25)	1.410	0.161
Blood urea nitrogen (mmol/L)	5.12 (3.18, 7.14)	4.96 (3.45, 6.84)	−0.224	0.823
Serum creatinine (μmol/L)	65.90 (56.50, 82.50)	69.00 (55.00, 83.00)	0.276	0.783
Creatine kinase (U/L)	220.00 (141.00, 398.00)	227.50 (137.00, 336.75)	0.105	0.917
Creatine kinase–MB (U/L)	13.60 (8.45, 20.00)	12.15 (6.25, 28.30)	0.415	0.678
Meningitis-consistent CSF abnormalities, *n* (%)	26 (53.06)	30 (26.79)	10.374	0.002
Early-onset neonatal sepsis, *n* (%)	39 (79.59)	76 (67.86)	2.300	0.184
Neonatal respiratory distress syndrome, *n* (%)	49 (100.00)	101 (90.18)	5.165	0.019
Patent ductus arteriosus, *n* (%)	31 (63.27)	64 (57.14)	0.528	0.491
Maternal and antenatal characteristics				
Age (years)	32.00 (28.00, 37.00)	33.00 (28.00, 36.00)	0.635	0.525
Cesarean section, *n* (%)	29 (59.18)	84 (75.00)	4.075	0.060
Antenatal corticosteroids, *n* (%)	40 (81.63)	90 (80.36)	0.036	0.850
Gestational hypertension, *n* (%)	11 (22.45)	39 (34.82)	2.437	0.141
Gestational diabetes mellitus, *n* (%)	8 (16.33)	26 (23.21)	0.971	0.404
Chorioamnionitis, *n* (%)	3 (6.12)	7 (6.25)	0.014	0.905
Premature rupture of membranes, *n* (%)	20 (40.82)	33 (29.46)	1.989	0.202
Multiple gestation, *n* (%)	11 (22.45)	27 (24.11)	0.052	0.820
Assisted reproductive technology, *n* (%)	16 (32.65)	26 (23.21)	1.575	0.243
Meconium-stained amniotic fluid, *n* (%)	2 (4.08)	9 (8.04)	0.837	0.506

Data are presented as median (Q1, Q3) for continuous variables and *n* (%) for categorical variables, as appropriate; values in parentheses for continuous variables indicate the first and third quartiles. Continuous variables were compared using the Mann–Whitney *U*-test, and categorical variables were compared using the χ^2^ test or Fisher's exact test, as appropriate. The χ^2^/Z value column reports χ^2^ values for categorical variables and Z values for continuous variables. Q1, first quartile; Q3, third quartile; IVH, Itraventricular hemorrhage; CSF, Cerebrospinal fluid.

## Results

### Baseline clinical characteristics

**Figure 1:**
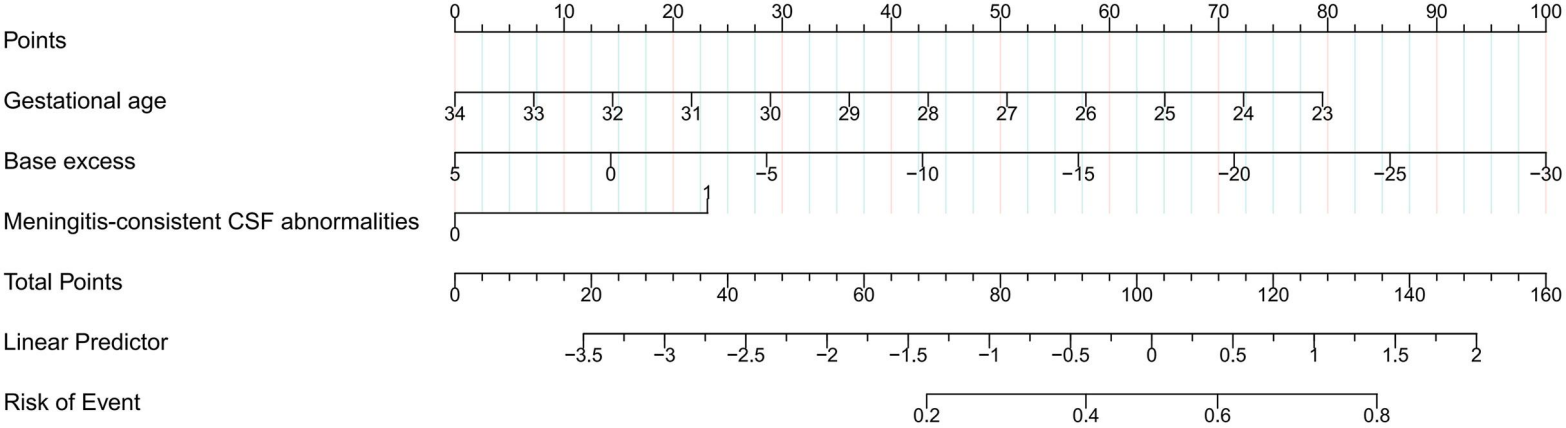
Flow diagram of participant selection across the three participating NICUs. IVH, intraventricular hemorrhage; NICU, neonatal intensive care unit.

A total of 169 very preterm and/or very low birth weight infants with IVH were assessed for eligibility across the three participating NICUs (Figure 1). Eight infants were excluded, including three with missing key predictor or outcome data and five in whom active treatment was withheld or withdrawn. Finally, 161 infants with complete data for candidate predictors and outcome assessment were included in the final analysis, including 49 with severe IVH and 112 with mild IVH. The general characteristics of the preterm infants and their mothers are summarized in Table 1. Several maternal and antenatal variables were compared between the severe and mild IVH groups, including chorioamnionitis, PROM, hypertensive disorders of pregnancy, gestational diabetes, antenatal corticosteroid exposure, multiple gestation, ART, and MSAF. None of these variables differed significantly between the two groups. All CSF cultures were negative; therefore, no infant met the definition based on positive CSF culture alone, and this variable was based on predefined CSF biochemical and cytological abnormalities rather than microbiologically confirmed meningitis. The median postnatal age at lumbar puncture was 2.0 (1.5, 2.3) days, whereas the median postnatal age at the first cranial ultrasound diagnosis of severe IVH was 3.3 (3.1, 3.6) days. In all infants included in the prediction model as having meningitis-consistent CSF abnormalities, CSF abnormalities were documented before or no later than the cranial ultrasound diagnosis of severe IVH.

Lumbar puncture was performed in 115 of the 161 infants (71.43%), including 39 of 49 infants (79.59%) in the severe IVH group and 76 of 112 infants (67.86%) in the mild IVH group. All infants who underwent lumbar puncture had clinically suspected or clinical EOS, and lumbar puncture was performed before or no later than the first cranial ultrasound diagnosis of severe IVH. Lumbar puncture was not performed in 46 infants because there was no clinical indication for CSF analysis based on the EOS evaluation framework. No infant with a clinical indication for lumbar puncture was documented as being unable to undergo CSF analysis solely because of persistent cardiorespiratory instability. Overall, 56 of 161 infants (34.78%) met the predefined criteria for meningitis-consistent CSF abnormalities. Among infants who underwent lumbar puncture, the corresponding proportion was 56 of 115 (48.70%), including 26 of 39 (66.67%) in the severe IVH group and 30 of 76 (39.47%) in the mild IVH group. Traumatic tap or visibly bloody CSF was documented in 2 of 115 infants who underwent lumbar puncture, including 1 of 39 in the severe IVH group and 1 of 76 in the mild IVH group. These data are summarized in Supplementary Table S3.

### Selection of perinatal characteristics

**Figure 2:**
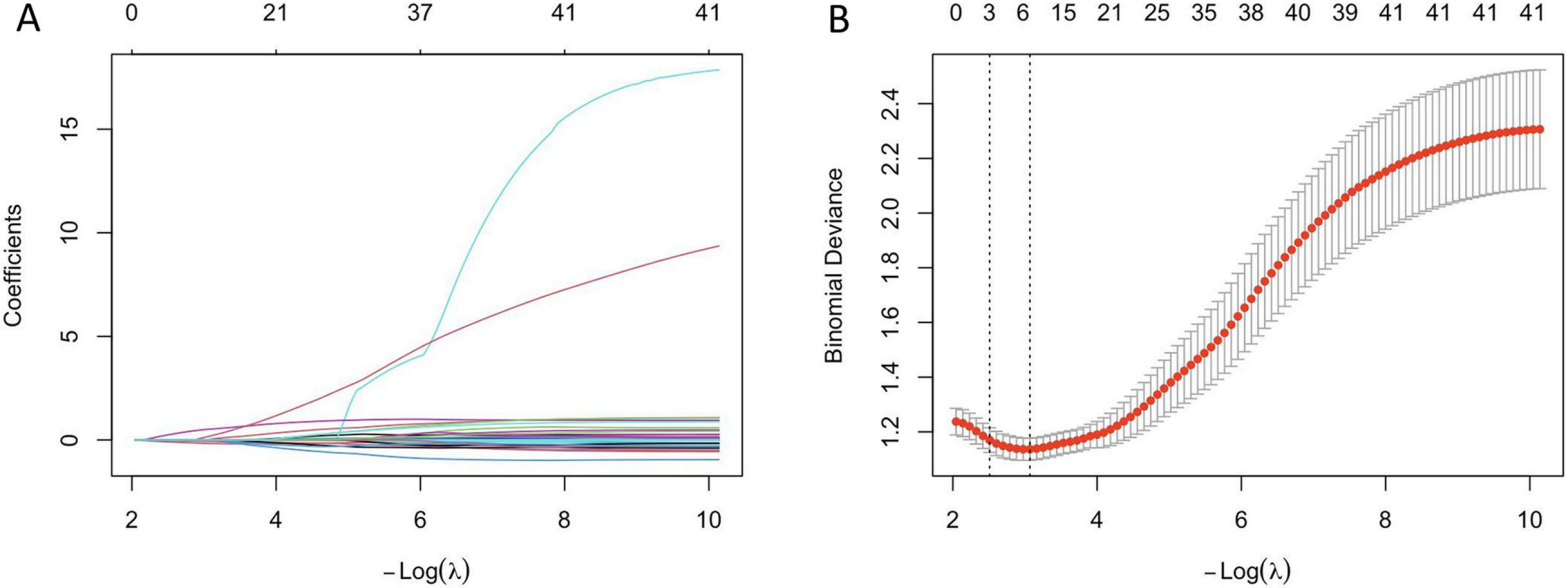
Selection of candidate predictors using LASSO regression. A total of 41 eligible candidate predictors listed in Table 1 and satisfying the temporal-ordering criterion were initially entered into the LASSO regression model. **(A)** Coefficient profiles of the candidate predictors. **(B)** Cross-validation was used to select the optimal penalty parameter. The vertical dotted line represents the optimal *λ*, which was 0.08124906 (log λ = −2.510236). LASSO, least absolute shrinkage and selection operator.

**Figure 3:**
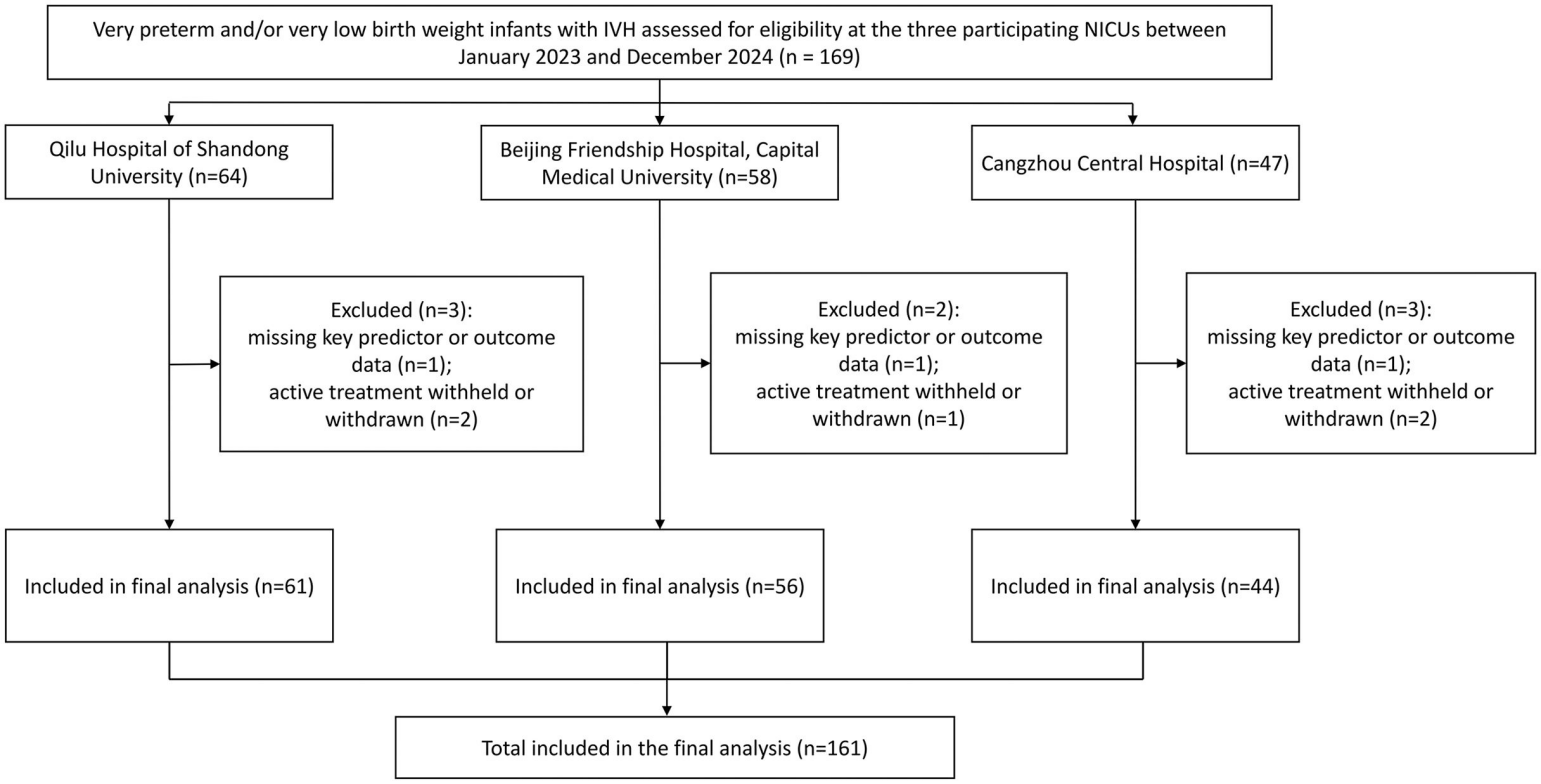
Nomogram for distinguishing severe from mild IVH based on gestational age, base excess, and meningitis-consistent CSF abnormalities. IVH, intraventricular hemorrhage; CSF, cerebrospinal fluid.

LASSO regression was used to select candidate predictors for severe IVH from the variables listed in Table 1 (Figures 2A,B). A total of 41 candidate predictors were initially entered into the LASSO regression model. As the tuning parameter λ changed, coefficients of less informative variables were shrunk toward zero, whereas variables with stronger predictive contributions retained nonzero coefficients. Using cross-validation, the optimal λ was determined to be 0.08124906 (log λ = −2.510236). LASSO regression retained three predictors with nonzero coefficients: gestational age at birth, base excess, and meningitis-consistent CSF abnormalities. These three variables were subsequently entered into the multivariable logistic regression model, and the nomogram was constructed accordingly (Table 2 and Figure 3).

**Table 2:** Predictive factors for distinguishing severe from mild IVH in very preterm and/or very low birth weight infants with IVH.

Variable	B	SE	Wald χ^2^	*P* value	OR (95% CI)
Gestational age	−0.304	0.102	8.802	0.003	0.738 (0.604, 0.902)
Base excess	−0.120	0.042	8.157	0.004	0.887 (0.817, 0.963)
Meningitis-consistent CSF abnormalities	0.971	0.381	6.505	0.011	2.642 (1.252, 5.573)

B, regression coefficient; SE, standard error; OR, odds ratio; CI, confidence interval; IVH, intraventricular hemorrhage; CSF, cerebrospinal fluid.

### Development of nomogram

A nomogram was developed based on the three predictors retained in the final multivariable logistic regression model: gestational age, base excess, and meningitis-consistent CSF abnormalities (Figure 3).

### Validation and performance of the prediction model

**Figure 4:**
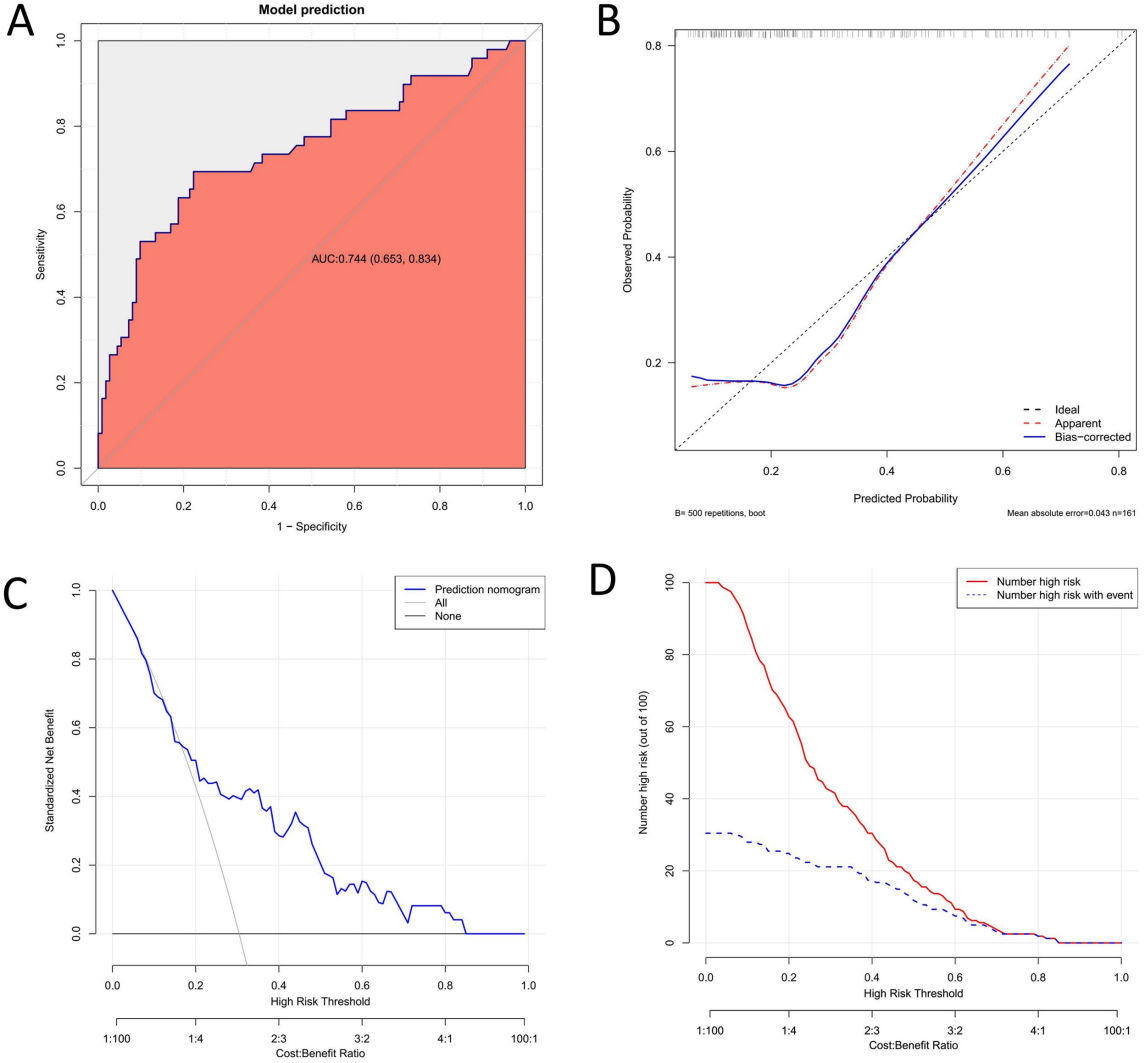
Model performance and exploratory clinical utility of the nomogram. **(A)** Receiver operating characteristic curve. **(B)** Calibration curve after bootstrap internal validation with 500 repetitions. **(C)** Exploratory decision curve analysis. **(D)** Exploratory clinical impact curve. IVH, intraventricular hemorrhage.

Model performance was evaluated using multiple complementary metrics. The apparent C-index/AUC was 0.744 (95% CI: 0.653–0.834), indicating moderate discrimination (Figure 4A). Bootstrap internal validation with 500 repetitions showed a mean optimism of 0.017, resulting in an optimism-corrected C-index/AUC of 0.727 (95% CI: 0.646–0.826). The apparent calibration intercept and slope were 0 and 1.000, respectively, while the optimism-corrected calibration intercept and slope were −0.036 and 0.934, respectively, suggesting some degree of optimism after internal validation. The Brier score was 0.171. The calibration plot showed apparent and bias-corrected curves with a mean absolute error of 0.043 (Figure 4B). Decision curve analysis and the clinical impact curve were used to explore potential clinical utility (Figures 4C,D). Because no external validation cohort was available, these findings should be interpreted as internally validated exploratory performance estimates rather than evidence of external generalizability.

## Discussion

In infants born at <32 weeks' gestation and/or with very low birth weight, prior studies consistently indicate that IVH remains a common complication, although reported rates vary substantially depending on gestational-age composition and inter-center differences. A systematic review and meta-analysis including studies published between 2010 and 2020 reported a pooled incidence of approximately 17.4% for IVH of any grade among infants born at 28–31 weeks, with severe IVH (Papile grades III–IV) occurring in about 4.6%. When cohorts were skewed toward <28 weeks, the incidence increased to roughly 34.3% for any-grade IVH and 15.0% for severe IVH, highlighting the progressively greater burden of severe hemorrhage with decreasing gestational age (8). This pattern has also been corroborated by recent prospective data; for example, in a cohort of 1,079 very preterm infants reported by Wang et al., the incidences of grade I–II and grade III–IV IVH were 35.2% and 6.9%, respectively (2). Collectively, these findings underscore that, in cohorts predominantly comprising infants <32 weeks and/or very low birth weight infants, IVH—particularly severe IVH—remains a clinically important risk, supporting the need for risk-stratification tools based on perinatal and early postnatal factors to facilitate earlier identification of high-risk infants and to optimize preventive and monitoring strategies.

In our study, we found that lower gestational age was associated with a higher risk of severe (grade III–IV) IVH. This observation is biologically plausible and can be explained by several converging pathophysiological mechanisms. First, IVH in very preterm infants typically originates from hemorrhage in the germinal matrix, a highly vascularized region in which the microvasculature is structurally immature. At lower gestational ages, the germinal matrix is more prominent, and its vessels have less developed supporting architecture (e.g., limited perivascular support and an incompletely mature barrier), making them particularly susceptible to rupture under shear stress and mechanical strain, with bleeding more likely to extend into the ventricular system (9, 10). Second, extremely preterm infants have immature cerebral blood flow autoregulation, and cerebral perfusion is therefore more “pressure-passive”. During the early postnatal transition, fluctuations in systemic blood pressure, PaCO_2_, oxygenation, and intravascular volume—as well as hemodynamic disturbances related to a patent ductus arteriosus and changes in venous return secondary to respiratory support—can provoke rapid swings in cerebral blood flow, triggering germinal matrix vessel rupture and promoting progression to ventricular dilatation and parenchymal involvement, thereby increasing the likelihood of severe IVH (11, 12). Finally, the relative immaturity of hemostatic and inflammatory control in preterm infants may further facilitate ongoing or progressive bleeding (13); inflammatory responses (e.g., infection-related) can exacerbate vascular fragility and barrier disruption, making early microbleeds less likely to stabilize and more prone to evolve into higher-grade hemorrhage (10). Taken together, lower gestational age reflects a combination of a more vulnerable anatomical substrate, greater hemodynamic instability, and immature coagulation/inflammatory regulation, which jointly contribute to the increased susceptibility to severe IVH.

In our cohort, a lower (more negative) BE was also associated with an increased risk of severe IVH. Physiologically, a markedly reduced BE generally indicates metabolic acidosis and/or inadequate tissue perfusion, which may occur in the context of perinatal hypoxia, hemodynamic instability, or a heightened inflammatory burden (14–16). These conditions can place the fragile germinal matrix vasculature under greater stress and, during early postnatal stabilization (e.g., resuscitation, volume expansion, vasoactive support, or ventilator adjustments), may amplify fluctuations in blood pressure, PaCO₂, and oxygenation (17, 18). Given the immature cerebral autoregulation in very preterm infants, such “pressure-passive” cerebral perfusion may predispose to vessel rupture and facilitate progression to ventricular dilatation and parenchymal involvement, thereby increasing the likelihood of severe IVH (19). Moreover, acidosis has been linked to impaired hemostasis (e.g., reduced thrombin activity, platelet dysfunction, and enhanced fibrinolysis), which may limit early stabilization of microbleeds and promote hemorrhage extension (20, 21). Notably, BE may also serve as a surrogate marker of overall illness severity and systemic hypoperfusion (22); therefore, its association with severe IVH may reflect both direct physiological effects and the broader burden of circulatory instability. Because BE was measured during the early postnatal period, it may represent not only a preceding risk indicator but also a marker of concurrent illness severity or evolving hemodynamic instability around the time of IVH development.

Although meningitis-consistent CSF abnormalities were documented before or no later than the first cranial ultrasound diagnosis of severe IVH in our cohort, cranial ultrasound detects IVH only at scheduled time points rather than at the exact biological onset of hemorrhage. Therefore, temporal ambiguity remains, and these CSF abnormalities should be interpreted as early clinical markers of inflammatory and illness burden, or potentially parallel manifestations of evolving severe IVH, rather than definitive causal predictors. Several biological mechanisms may explain this association. First, infection-related systemic and central nervous system inflammation may aggravate the inherent fragility of the germinal matrix vasculature in very preterm and/or very low birth weight infants, which is characterized by immature basement membranes, limited pericyte coverage, and poor structural support (23). Second, evidence from bacterial meningitis suggests that central nervous system inflammation may impair blood–brain barrier integrity and damage brain microvascular endothelial cells, thereby increasing vascular permeability and amplifying neuroinflammation (24). Third, sepsis-associated coagulopathy, including thrombocytopenia and consumption of coagulation factors, may further increase bleeding susceptibility (25). Finally, systemic infection or central nervous system inflammation may be accompanied by respiratory and hemodynamic instability, which can disturb cerebral autoregulation and expose the fragile preterm cerebral vasculature to abrupt fluctuations in cerebral blood flow (26). Taken together, these pathways may partly explain why infants with meningitis-consistent CSF abnormalities showed a higher risk of severe IVH in our study. It should also be noted that meningitis-consistent CSF abnormalities in the first days of life may reflect not only postnatal central nervous system infection, but also an antenatal or perinatal inflammatory process. Intrauterine inflammation may contribute to both early-onset infection and increased vulnerability of the germinal matrix vasculature through systemic cytokine activation, endothelial injury, and hemodynamic instability. In addition, CSF parameters in extremely preterm infants may be influenced by systemic inflammation, traumatic lumbar puncture, blood contamination, or evolving IVH itself, which may complicate the biological interpretation of this variable.

Although the three final predictors—gestational age, BE, and meningitis-consistent CSF abnormalities—are clinically recognizable markers of prematurity and illness severity, the nomogram may still provide added practical value by integrating these variables into a single quantitative and visual risk-estimation tool. Rather than replacing clinical judgment, the model may help standardize preliminary risk stratification, support communication among clinicians, and identify infants with IVH who may warrant closer hemodynamic monitoring and repeat cranial ultrasound surveillance. However, we emphasize that this potential clinical value remains exploratory. The present nomogram has not been externally validated and has not been tested in an impact study; therefore, it should not be used as a definitive decision rule or as a substitute for standard neonatal assessment.

Several limitations should be acknowledged. First, this was a retrospective study based on routinely recorded clinical data. Therefore, unmeasured confounding, potential misclassification, and center-specific documentation differences could not be completely excluded. Although several maternal and antenatal variables were collected, more detailed antenatal inflammatory indicators, such as maternal intrapartum fever, histological chorioamnionitis, funisitis, and placental pathological findings, were not consistently available. Second, the sample size was relatively limited, particularly in relation to the number of severe IVH events, which may have affected model stability and increased the risk of overfitting. This concern is especially relevant because meningitis-consistent CSF abnormalities had an unexpectedly high prevalence in this cohort and may have influenced variable selection and model performance. Third, although predictors were selected only when documented before or no later than the first cranial ultrasound diagnosis of severe IVH, the exact biological onset of IVH likely preceded the first cranial ultrasound detection in some infants. Therefore, temporal ambiguity cannot be fully excluded. IVH occurring outside this early surveillance window or subtle interval progression between examinations may have been missed or classified later than its true onset. Thus, some early postnatal markers, including BE and CSF abnormalities, may reflect concurrent or evolving pathophysiological processes rather than purely antecedent predictors. Fourth, meningitis-consistent CSF abnormalities should be interpreted cautiously because lumbar puncture was performed only in infants with clinically suspected or clinical EOS rather than routinely in all infants. Although no infant with a clinical indication for lumbar puncture was documented as being unable to undergo CSF analysis solely because of persistent instability, indication-related selection bias and CSF-related misclassification cannot be excluded. All CSF cultures were negative, and CSF parameters may be influenced by systemic illness, traumatic taps, blood contamination, or evolving IVH itself. Fifth, missing data were handled using complete-case analysis, which may have introduced selection bias if missingness was related to illness severity or center-specific documentation practices. Sixth, decision curve analysis and the clinical impact curve were exploratory because they were derived from the development cohort and were not externally validated. Seventh, because the analytic cohort included only infants with IVH, the present model was designed to distinguish severe from mild IVH rather than to predict the initial occurrence of IVH among all very preterm and/or very low birth weight infants. Finally, according to the hierarchy of prediction model validation, the present study represents model development with internal validation only. Although bootstrap resampling was used to assess optimism and calibration, external validation, recalibration if necessary, and clinical impact analysis are still required before clinical implementation.

## Conclusions

In summary, we developed an exploratory nomogram based on a small set of routinely obtainable clinical variables to distinguish severe IVH from mild IVH among very preterm and/or very low birth weight infants with IVH. The model showed moderate discrimination and acceptable apparent calibration in this retrospective cohort. This nomogram may help preliminary risk stratification and identify infants who may warrant closer cranial ultrasound surveillance and monitoring. However, larger external validation studies are needed before broader clinical implementation.

## Data Availability

The raw data supporting the conclusions of this article will be made available by the authors, without undue reservation.

## References

[1] Nagy Z Obeidat M Máté V Nagy R Szántó E Veres DS. Occurrence and time of onset of intraventricular hemorrhage in preterm neonates: a systematic review and meta-analysis of individual patient data. JAMA Pediatr. (2025) 179(2):145–54. 10.1001/jamapediatrics.2024.5998 39786414 PMC11791718

[2] Wang Y Song J Zhang X Kang W Li W Yue Y. The impact of different degrees of intraventricular hemorrhage on mortality and neurological outcomes in very preterm infants: a prospective cohort study. Front Neurol. (2022) 13:853417. 10.3389/fneur.2022.853417 35386416 PMC8978798

[3] Tréluyer L Chevallier M Jarreau P Baud O Benhammou V Gire C. Intraventricular hemorrhage in very preterm children: mortality and neurodevelopment at age 5. Pediatrics. (2023) 151(4):e2022059138. 10.1542/peds.2022-059138 36919442 PMC10071431

[4] Rees P Callan C Chadda KR Vaal M Diviney J Sabti S. Preterm brain injury and neurodevelopmental outcomes: a meta-analysis. Pediatrics. (2022) 150(6):e2022057442. 10.1542/peds.2022-057442 36330752 PMC9724175

[5] Schüssler SC Paul A Niederreiter U Deiters L Fahlbusch FB Morhart P. Seizures in preterm infants with germinal-matrix-intraventricular hemorrhage (GM-IVH): a retrospective monocentric study on predictors and neurodevelopmental outcome. Eur J Paediatr Neurol. (2025) 56:51–7. 10.1016/j.ejpn.2025.04.012 40311512

[6] Bell EF Hintz SR Hansen NI Bann CM Wyckoff MH Demauro SB. Mortality, in-hospital morbidity, care practices, and 2-year outcomes for extremely preterm infants in the US, 2013–2018. JAMA. (2022) 327(3):248–63. 10.1001/jama.2021.23580 35040888 PMC8767441

[7] Cai Y Li X Zhao X Song Y Zhou W. Risk assessment and early prediction of intraventricular hemorrhage in extremely preterm infants. Sci Rep. (2025) 15(1):17346. 10.1038/s41598-025-02061-4 40389542 PMC12089482

[8] Lai GY Shlobin N Garcia RM Wescott A Kulkarni AV Drake J. Global incidence proportion of intraventricular haemorrhage of prematurity: a meta-analysis of studies published 2010–2020. Arch Dis Child. (2022) 107(5):513–9. 10.1136/archdischild-2021-322634 34930831

[9] Egesa WI Odoch S Odong RJ Nakalema G Asiimwe D Ekuk E. Germinal matrix-intraventricular hemorrhage: a tale of preterm infants. Int J Pediatr. (2021) 2021:6622598. 10.1155/2021/6622598 33815512 PMC7987455

[10] Dermitzaki N Baltogianni M Tsiogka CM Nikolaou A Balomenou F Giapros V. Promising preventive strategies for intraventricular hemorrhage in preterm neonates: a critical review. J Clin Med. (2025) 14(19):6763. 10.3390/jcm14196763 41095842 PMC12524917

[11] Lahr BE Brunsch CL Dikkers R Bos AF Kooi EMW. Cerebrovascular autoregulation in preterm infants using heart rate or blood pressure: a pilot study. Children. (2024) 11(7):765. 10.3390/children11070765 39062215 PMC11276379

[12] Piscopo BR Sutherland AE Malhotra A Allison BJ Miller SL. Pathogenesis of preterm intraventricular haemorrhage. Dev Neurosci. (2026) 48:225–36. 10.1159/000546607 40451162

[13] Jiang L Yu Q Li H Wang F Liu F Xu Z. Blood pressure variability combined with coagulation function in early prediction and outcome assessment of germinal matrix-intraventricular hemorrhage in preterm infants with gestational age ≤ 32 weeks. PLoS One. (2025) 20(7):e0328904. 10.1371/journal.pone.0328904 40705748 PMC12289036

[14] Dhugga G Sankaran D Lakshminrusimha S. ABCs of base therapy in neonatology: role of acetate, bicarbonate, citrate and lactate. J Perinatol. (2025) 45(3):298–304. 10.1038/s41372-024-02169-x 39533025 PMC11888986

[15] Agakidou E Chatziioannidis I Kontou A Stathopoulou T Chotas W Sarafidis K. An update on pharmacologic management of neonatal hypotension: when, why, and which medication. Children. (2024) 11(4):490. 10.3390/children11040490 38671707 PMC11049273

[16] Zander R. Base excess (be): reloaded. Eur J Med Res. (2024) 29(1):281. 10.1186/s40001-024-01796-6 38735983 PMC11089692

[17] Goswami IR Abou Mehrem A Scott J Esser MJ Mohammad K. Metabolic acidosis rather than hypo/hypercapnia in the first 72 h of life associated with intraventricular hemorrhage in preterm neonates. J Matern Fetal Neonatal Med. (2021) 34(23):3874–82. 10.1080/14767058.2019.1701649 31852289

[18] Ćaleta T Ryll MJ Bojanić K Dessardo NSI Schroeder DR Sprung J. Regional cerebral oxygen saturation variability and brain injury in preterm infants. Front Pediatr. (2024) 12:1426874. 10.3389/fped.2024.1426874 39105161 PMC11298368

[19] Pal A Stewart D Ojha K Kriss V Ziegler C Fischer H. Cerebral resistive indices and intraventricular hemorrhage in premature neonates <29 weeks’ gestation: a pilot prospective cohort study. BMC Pediatr. (2025) 25(1):766. 10.1186/s12887-025-06119-0 41044658 PMC12495792

[20] Shimono K Ito T Kamikokuryo C Niiyama S Yamada S Onishi H. Damage-associated molecular patterns and fibrinolysis perturbation are associated with lethal outcomes in traumatic injury. Thromb J. (2023) 21(1):91. 10.1186/s12959-023-00536-w 37674235 PMC10481518

[21] Moore EE Moore HB Kornblith LZ Neal MD Hoffman M Mutch NJ. Trauma-induced coagulopathy. Nat Rev Dis Primers. (2021) 7(1):30. 10.1038/s41572-021-00264-3 33927200 PMC9107773

[22] Kumar Krishnegowda V Prasath A Vadakkencherry Ramaswamy V Trevisanuto D. Neonatal shock: current dilemmas and future research avenues. Children. (2025) 12(2):128. 10.3390/children12020128 40003230 PMC11854444

[23] Atienza-Navarro I Alves-Martinez P Lubian-Lopez S Garcia-Alloza M. Germinal matrix-intraventricular hemorrhage of the preterm newborn and preclinical models: inflammatory considerations. Int J Mol Sci. (2020) 21(21):8343. 10.3390/ijms21218343 33172205 PMC7664434

[24] Yang R Wang J Wang F Zhang H Tan C Chen H. Blood-brain barrier integrity damage in bacterial meningitis: the underlying link, mechanisms, and therapeutic targets. Int J Mol Sci. (2023) 24(3):2852. 10.3390/ijms24032852 36769171 PMC9918147

[25] Aziz KB Saxonhouse M Mahesh D Wheeler KE Wynn JL. The frequency and timing of sepsis-associated coagulopathy in the neonatal intensive care unit. Front Pediatr. (2024) 12:1364725. 10.3389/fped.2024.1364725 38504996 PMC10948397

[26] Tsao PC. Pathogenesis and prevention of intraventricular hemorrhage in preterm infants. J Korean Neurosurg Soc. (2023) 66(3):228–38. 10.3340/jkns.2022.0288 36919227 PMC10183267

