# Supplementary material for: Predicting severe intraventricular hemorrhage in very preterm and/or very low birth weight infants: a nomogram approach
Source: Front Pediatr. 2026 Jun 3;14:1838932. doi: 10.3389/fped.2026.1838932 (PMC13272528; doi:10.3389/fped.2026.1838932)
Supplement: Supplementary file 1 [file Table1.docx]

Supplementary Table S1. Diagnostic criteria for early-onset sepsis (EOS)

EOS was classified into suspected, clinical, and confirmed categories as follows:

1. Suspected EOS: Infants with clinical abnormalities within ≤72 hours after birth or the presence of major high-risk factors for EOS.

2. Clinical EOS: Infants with clinical abnormalities, plus any one of the following criteria: (1) ≥2 positive findings on nonspecific blood tests; (2) cerebrospinal fluid (CSF) abnormalities consistent with meningitis; (3) Detection of pathogen DNA in blood or CSF.

3. Confirmed EOS: Infants with compatible clinical manifestations, with positive cultures from blood or CSF (or other sterile body fluids).

In this study, CSF findings and pathogen DNA detected in CSF were not used to establish EOS eligibility; EOS eligibility was determined based on clinical manifestations within 72 hours plus ≥2 abnormal nonspecific blood tests.

Clinical-specific manifestations included the following:

Systemic signs: temperature instability not attributable to environmental factors (fever ≥38 °C or hypothermia <36 °C).

Respiratory system: tachypnea (respiratory rate ≥60 breaths/min), apnea, and cyanosis. In EOS, apnea or respiratory distress may be the initial manifestation and persist for >6 hours.

Cardiovascular system: persistent tachycardia (heart rate ≥160 beats/min) or persistent bradycardia (heart rate <100 beats/min), pallor, cold extremities, mottled (marbled) skin, hypotension, or capillary refill time >3 seconds.

Skin: ashen/grayish complexion, skin and/or lip cyanosis, ecchymosis, or petechiae.

Neurologic system: seizures, bulging anterior fontanelle, hypotonia, lethargy, and poor responsiveness.

Gastrointestinal system: poor feeding or feeding refusal, abdominal distension, vomiting or gastric residuals, diarrhea, and hepatosplenomegaly.

Urinary system: oliguria and renal failure.

Hematologic system: bleeding tendency, purpura, coagulopathy, and unexplained jaundice.

Non-specific blood test abnormalities were defined as follows:

1. White blood cell (WBC) count: abnormal if ≥25 × 10⁹/L between 6 h and 3 days of life, or <5 × 10⁹/L at any postnatal age, or if the absolute neutrophil count (ANC) <1 × 10⁹/L.

2. Immature-to-total neutrophil ratio (I/T ratio): abnormal if I/T ≥0.16 from birth to 3 days of life.

3. C-reactive protein (CRP): abnormal if ≥3 mg/L within 0–6 h after birth, ≥5 mg/L at 6–24 h, or ≥10 mg/L after 24 h of life.

4. Procalcitonin (PCT): abnormal values were interpreted according to postnatal age, defined as ≥0.5 μg/L within 0–6 h, ≥2 μg/L at 6–12 h and 48–60 h, ≥5 μg/L at 12–18 h and 36–48 h, ≥10 μg/L at 18–36 h, and ≥1 μg/L at 60–72 h after birth.

Supplementary Table S2. Definitions and timing of key variables

| **Variable** | **Definition** | **Timing/source** |
| --- | --- | --- |
| Severe IVH | Papile grade III–IV | Cranial ultrasound on days 1–3 and 5–7 |
| BE | Base excess from arterial blood gas | First arterial blood gas after NICU admission and within 24 h after birth |
| Meningitis-consistent CSF abnormalities | CSF WBC ≥20/mm³ with >50% PMNs plus hypoglycorrhachia, with or without markedly elevated CSF protein (>1880 mg/L), or positive CSF culture | LP when clinically indicated and feasible; before or no later than first severe IVH diagnosis |
| EOS | Clinical manifestations within 72 h plus ≥2 abnormal nonspecific blood tests | Within 72 h after birth; detailed criteria in Supplementary Table S1 |
| Candidate predictors | Eligible variables in Table 1 satisfying the temporal-ordering criterion | Initially entered into LASSO |

IVH, intraventricular hemorrhage; BE, base excess; CSF, cerebrospinal fluid; WBC, white blood cell; PMNs, polymorphonuclear leukocytes; LP, lumbar puncture; EOS, early-onset sepsis; LASSO, least absolute shrinkage and selection operator.

Supplementary Table S3. Lumbar puncture, CSF assessment, and missing-data summary

| **Variable** | **Overall n=161** | **Severe IVH n=49** | **Mild IVH n=112** |
| --- | --- | --- | --- |
| **Lumbar puncture clinically indicated based on suspected/clinical EOS** | **115 (71.43%)** | **39 (79.59%)** | **76 (67.86%)** |
| **Lumbar puncture performed** | **115 (71.43%)** | **39 (79.59%)** | **76 (67.86%)** |
| **Lumbar puncture not performed because CSF analysis was not clinically indicated** | **46 (28.57%)** | **10 (20.41%)** | **36 (32.14%)** |
| **Lumbar puncture not performed because of persistent cardiorespiratory instability despite indication** | **0** | **0** | **0** |
| **Meningitis-consistent CSF abnormalities in total cohort** | **56/161 (34.78%)** | **26/49 (53.06%)** | **30/112 (26.79%)** |
| **Meningitis-consistent CSF abnormalities among infants with LP** | **56/115 (48.70%)** | **26/39 (66.67%)** | **30/76 (39.47%)** |
| **Positive CSF culture among infants with LP** | **0/115 (0%)** | **0/39 (0%)** | **0/76 (0%)** |
| **Traumatic tap / visibly bloody CSF** | **2/115 (1.74%)** | **1/39 (2.56%)** | **1/76 (1.32%)** |
| **Missing key predictor or outcome data among eligible infants** | **3/169 (1.8%)** | **—** | **—** |

IVH, intraventricular hemorrhage; EOS, early-onset sepsis; CSF, cerebrospinal fluid; LP, lumbar puncture.
